# The prognostic value of baseline erosions in undifferentiated arthritis

**DOI:** 10.1186/ar2832

**Published:** 2009-10-15

**Authors:** Mohamed M Thabet, Thomas WJ Huizinga, Désirée M van der Heijde, Annette HM van der Helm-van Mil

**Affiliations:** 1 Department of Rheumatology, Leiden University Medical Center, Albinusdreef 2, Leiden, PO Box 9600, 2300RC, The Netherlands; 2 Department of Internal Medicine, Assiut University Hospital, University Street 1, Assiut, P.O. Box 71515, Egypt

## Abstract

**Introduction:**

Undifferentiated arthritis (UA) has a variable disease course; 40 to 50% of UA patients remit spontaneously, while 30% develop rheumatoid arthritis (RA). Identifying the UA patients who will develop RA is essential to initiate early disease-modifying anti-rheumatic drug (DMARD) therapy. Although the presence of bone erosions at baseline is predictive for a severe destructive disease course in RA, the prognostic importance of erosive joints for disease outcome in UA is unknown. This study evaluates the predictive value of erosive joints for the disease outcome in UA as measured by RA development and disease persistency.

**Methods:**

Baseline hands and feet radiographs of 518 UA patients were evaluated for erosions using a clinical definition as well as the Sharp/van der Heijde method. After 1 year follow-up, patients were re-assessed for the fulfilment of the 1987 ACR classification criteria for RA. Disease persistency was defined as the absence of sustained remission during all available follow-up (mean 8 ± 3 years).

**Results:**

At baseline, 28.6% of UA patients had erosive joints. Presence of ≥2 erosive joints showed a positive predictive value for RA development of 53% and for persistent disease of 68%. Patients with erosions that did not develop RA were less often anticyclic citrullinated peptide antibody (ACPA)+ve, rheumatoid factor (RF)+ve and had lower C-reactive protein (CRP), erythrocytic sedimentation rate (ESR) and number of swollen joints compared to those who developed RA. Feet erosions are equally predictive compared to erosions at hands.

**Conclusions:**

Presence of ≥2 erosive joints at baseline in UA patients gives a risk for RA development of 53% and for persistent disease of 68%, indicating that erosions in UA are not always predictive for unfavorable disease outcomes.

## Introduction

Early undifferentiated arthritis (UA) is defined as any arthritis of recent onset that cannot be classified according to the existing criteria for specific rheumatic disorders [1]. Patients with early UA form a heterogeneous group exhibiting a variable disease course. Of patients with early UA, 40 to 50% remit spontaneously, whereas 30% develop rheumatoid arthritis (RA) [2-4]. Recent data indicate that initiation of disease-modifying anti-rheumatic drug (DMARD) therapy in an early stage is beneficial and thus underlines the necessity to recognize those UA patients that will develop RA [5].

In the clinical setting generally much impact is given to clinical predictors of the disease outcome. In RA the early occurrence of erosions is one of the most significant predictors for a severe destructive disease course. In contrast, the prognostic value of baseline erosions for the disease outcome in UA is unknown. Even more, the definition of erosive disease is unclear and different studies use different descriptions and cut-off values. Interestingly, the presence of erosions is part of the 1987 American College of Rheumatology (ACR) classification criteria for RA, but it is not specified how many erosions are required and it only includes erosions in the hands and not in the feet [6].

Considering the lack of knowledge on the prognostic value of erosions in UA, the present study aims to: study the predictive value of erosive joints in hands and feet for development of RA in UA-patients; define the optimal number of erosive joints to predict RA; define whether the predictive ability is different between erosive joints in hands and feet; determine whether information on erosive joints increases the discriminative ability of a recently developed prediction rule for RA-development [7,8]; and investigate whether the results are different when disease persistency is studied instead of the development of RA according to the 1987 ACR criteria.

The presence of erosions was assessed using two methods. First, as the present study aims to have results that are useful for clinical practice, we defined an erosive joint as a joint with at least one erosion, defined as a lesion with an interrupted cortex. The number of erosive joints was counted. As such, this definition is the same as the erosion score in the Simplified Erosion Narrowing Score (SENS) and is in line with common clinical practice [9]. Several other scoring methods quantify the radiological joint destruction in more detail [10]. Commonly used are the Sharp and the Larsen methods with their modifications [11,12]. Although these methods are very valuable for research purposes, they are difficult to use in clinical practice [10] because they require specialized training and are time-consuming [13,14]. We primarily studied the predictive value of the number of erosive joints but also performed the analyses using the erosion score of the Sharp/van der Heijde scoring (SHS) method in which the size and number of erosions per joint are weighted [15].

## Materials and methods

### Patients

The present study includes 518 early arthritis patients who were included between 1993 and 2005 in the Leiden Early Arthritis Cohort (EAC). This EAC is a prospective cohort started in 1993 [16]. Patients were referred by general practitioners when arthritis was suspected and inclusion took place when arthritis was confirmed at physical examination and symptom duration was less than two years. Written informed consent was obtained from all participants. The study was approved by the local Medical Ethical Committee. At inclusion, patients were asked about their joint symptoms, disease duration and subjected to a physical examination. Blood samples were taken for routine diagnostic laboratory screening (including immunoglobulin (Ig)M-rheumatoid factor (RF)) and stored to determine other autoantibodies at a later time. Follow-up visits were performed on a yearly basis and included radiographs of hands and feet. Patients who at baseline did not fulfil the criteria for known rheumatic disorders and thus were referred to as having UA (518 patients).

### Radiographs

Baseline radiographs of the hands (anterioposterior view) and feet (posterioanterior view) were available for all the 518 UA patients and were evaluated by the same person (MT). Erosions were defined according to the erosive score of the SENS method that was developed for use in clinical practice, by the presence of a joint with at least one erosion. Subsequently, the number of erosive joints was counted in the following joints: in hands, the proximal inter-phalangeal (PIP) joint in digits 1 to 5, the metacarpo-phalangeal (MCP) joint in digits 1 to 5 and 6 radio-carpal sites (base of metacarpal bone digit 1, trapezium, lunate, scaphoid, distal ulna and distal radius) and in feet, the inter-phalangeal (IP) joint digit 1 and metatarso-phalangeal (MTP) joint in digits 1 to 5.

In addition, the radiographs were scored according to the SHS method [15] assessing the same hand and feet joints as the SENS method. In the present study, only the erosion scores were studied while the joint space narrowing scores were omitted. Of the radiographs, 10% were scored twice to determine the intra-class correlation coefficient (ICC). With 10% rescoring, the erosive joint count according to the SENS definition showed an ICC of 0.91 and a smallest detectable change (SDC) of 0.92, while the erosion SHS scores showed an ICC of 0.94 and an SDC of 1.04.

### Disease outcome

All patients were followed prospectively. After one year of follow up, the fulfilment of the 1987 ACR criteria for RA was evaluated. The second disease outcome was disease persistency. As a generally accepted definition for persistency is lacking, we defined disease persistency as the absence of sustained remission during all available follow up (mean 8 ± 3 years). Sustained remission was diagnosed when patients had no swollen joints for at least one year, after cessation of eventual DMARD therapy. The absence of swollen joints had to have been observed by a rheumatologist for at least one year to ensure that remission was not temporary, but rather sustained. Most patients identified with remission were discharged from the outpatient clinic. Although patients should have absence of arthritis for at least one year according to the definition, most patients with remission had a longer follow up after the identification of remission (median of 16 months). Patients who had a recurrence of their arthritis after discharge, could easily return to the Leiden University Medical Center, the only referral center for rheumatology in a health care region of approximately 400,000 inhabitants. This occurred in six patients and these patients were not classified as sustained remission.

### Statistical analysis

Proportions were compared using the chi-squared test with two degrees of freedom (Epi Info v6, CDC, Atlanta, Georgia, USA). Differences in mean values between groups were analyzed with the Mann-Whitney U test. The positive predictive values (PPV), negative predictive values (NPV), specificity, sensitivity, and positive and negative likelihood ratios (LR) were determined for several cut-off values of the erosive joint count and erosion scores according to SHS method. Receiver operating characteristic (ROC) curves for the different cut-off values were constructed and the area under the curve (AUC) provided a measure of the overall discriminative ability. SPSS software, version 14.0 (Chicago, IL, USA), was used for data analyses. *P *values less than 0.05 were considered statistically significant.

## Results

### Predictive accuracy for RA development

From the 518 UA patients, 31% (n = 160) fulfilled the 1987 ACR classification criteria for RA after one year of follow-up. Baseline characteristics of UA patients that did and did not progress to RA are summarized in Table 1.

**Table 1:** Baseline characteristics of the total population of UA patients and for the patients that did and did not develop RA within one year

Characteristic	Total (n = 518)	Developed RA (n = 160)	Did not develop RA (n = 358)	*P*	LR+	LR-
**Female gender n (%)**	305 (59)	111 (69)	194 (54)	0.001	1.3	0.67
**Age in years (Mean ± SD)**	50.6 ± 16.5	56.3 ± 15	48 ± 16.5	< 0.001	-	-
**Symptom duration at inclusion in months (Mean ± SD)**	4.9 ± 5.5	5.9 ± 6	4.4 ± 5.1	0.002	-	-
**Morning stiffness (Mean ± SD)**	55.7 ± 89.9	82.9 ± 111.4	43.6 ± 75.6	< 0.001	-	-
**Tender joint count (Mean ± SD)**	6.5 ± 6.3	9.5 ± 7.4	5.1 ± 5.3	< 0.001	-	-
**Swollen joint count (Mean ± SD)**	3.8 ± 4	5.7 ± 5.1	2.9 ± 3.1	< 0.001	-	-
**HAQ score (Mean ± SD)**	0.76 ± 0.6	0.9 ± 0.6	0.7 ± 0.6	< 0.001	-	-
**ESR (mm/hr) (Mean ± SD)**	28.9 ± 24.4	36.8 ± 23.7	25.3 ± 23.9	< 0.001	-	-
**CRP (mg/L) (Mean ± SD)**	20.6 ± 28.2	26.5 ± 29.1	17.9 ± 27.4	< 0.001	-	-
**RF positivity n (%)**	125 (24.2)	76 (47.5)	49 (13.7)	< 0.001	3.5	0.61
**ACPA positivity n (%)**	114 (23.4)	76 (50)	38 (11.3)	< 0.001	4.4	0.56
**Having erosive joints in hands and/or feet n (%)	148 (28.6)	67 (41.9)	81 (22.6)	< 0.001*	1.8	0.75
**Hands only n (%)	58 (11.2)	23 (14.4)	35 (9.8)	0.1	-	-
**Feet only n (%)	46 (8.9)	22 (13.8)	24 (6.7)	0.009	-	-
**Hands and feet n (%)	44 (8.5)	22 (13.8)	22 (6.2)	0.004	-	-
**SHS erosion score (mean ± SD)**	0.8 ± 2	1.5 ± 3	0.5 ± 1.3	< 0.001	-	-

ACPA = anticyclic citrullinated peptide antibody; CRP = C-reactive protein; ESR = erythrocytic sedimentation rate; HAQ = health assessment questionnaire; LR+ = positive likelihood ratio; LR- = negative likelihood ratio; RA = rheumatoid arthritis; RF = rheumatoid factor; SD = standard deviation; SHS = Sharp/van der Heijde scoring; UA = undifferentiated arthritis.

Morning stiffness score on a 100 mm visual analogue scale.

* *P *value is calculated in reference to the no-erosion group; ** Defined as a broken cortex in at least 1 joint.

Overall, 148 of 518 (28.6%) UA patients had erosive joints at baseline. Seventy-six of the 160 (42%) patients who developed RA had baseline erosive joints, compared with 81 of the 358 (22.6%) who did not develop RA (*P *≤ 0.001).

Different cut-off values for erosive joint count were tested for their PPV and NPV regardless the localization of erosive joints (Table 2). The PPV of having one or more erosive joint was 45%. With higher cut-offs the PPV gradually increased. In the presence of two or more, three of more and four or more erosive joints the PPVs were roughly similar: 53 to 54%. For the cut-off of having five or more erosive joints, the PPV was 73%. As the 95% confidence interval (CI) were overlapping, it could not be concluded that the PPV of one of these cut-off values is statistically superior to the others. The specificity was significantly different between the cut-off of two or more erosive joints (89%) compared with the cut-off of one or more erosive joints (77%). Using higher cut-off values, the specificity gradually increased but the 95% CI overlapped. Evaluating the positive and negative LR revealed the cut-off of two or more erosive joints had the best balance between the positive and negative LRs.

**Table 2:** Predictive value for the progression from UA to RA within one year using different cut-off values for erosions in hands and/or feet

Number of erosive joints	n	PPV	(95% CI)	NPV	(95% CI)	Specificity	(95% CI)	Sensitivity	(95% CI)	LR+	LR-	AUC (SEM)
**≥1**	148	45	37-53	75	70-79	77	73-82	42	34-50	1.8	0.75	0.60 (0.028)
**≥2**	83	53	42-64	73	69-78	89	86-92	28	21-34	2.5	0.81	0.58 (0.028)
**≥3**	50	54	40-68	72	68-76	94	91-96	17	11-23	2.6	0.89	0.55 (0.028)
**≥4**	24	54	34-74	70	66-74	97	95-99	8	4-12	2.6	0.95	0.53 (0.028)
**≥5**	15	73	51-96	70	66-74	99	98-100	7	3-11	6.2	0.94	0.53 (0.028)

n = number of UA patients positive for this cut off.

AUC = area under the curve; CI = confidence interval; LR+ = positive likelihood ratio; LR- = negative likelihood ratio; NPV = negative predictive value; PPV = positive predictive value; RA = rheumatoid arthritis; SEM = standard error of measurement; UA = undifferentiated arthritis.

Additionally, the predictive values of the SHS erosion score were investigated in a similar way, using different cut-off values (Table 3). In case of an erosion score of one ore more, 45% of UA patients developed RA, which increased to 51 and 61% of UA patients in the presence of an erosion score of two or more and five or more, respectively. As such, the resulting PPVs and NPVs were comparable with those obtained using the erosive joint count. Also, here the cut off of an erosion score of two had the best balance between the positive and negative LRs.

**Table 3:** Predictive value for the progression from UA to RA within one year using the erosion score of the Sharp van der Heijde method with different cut-off values for erosions

SHS erosion score	n	PPV	(95% CI)	NPV	(95% CI)	Specificity	(95% CI)	Sensitivity	(95% CI)	LR+	LR-	AUC (SEM)
**≥1**	148	45	37-53	75	70-79	77	73-82	42	34-50	1.9	0.75	0.60 (0.028)
**≥2**	92	51	41-61	73	69-78	87	84-91	29	22-36	2.3	0.80	0.58 (0.028)
**≥3**	61	52	40-65	72	68-76	92	89-95	20	14-26	2.5	0.87	0.56 (0.028)
**≥4**	39	56	41-72	71	67-75	95	93-98	14	8-19	2.9	0.90	0.55 (0.028)
**≥5**	23	61	41-81	71	67-75	97	96-99	9	4-13	3.5	0.94	0.53 (0.028)

n = number of UA patients positive for this cutoff.

AUC = area under the curve; CI = confidence interval; LR+ = positive likelihood ratio; LR- = negative likelihood ratio; NPV = negative predictive value; PPV = positive predictive value; RA = rheumatoid arthritis; SEM = standard error of measurement; SHS = Sharp/van der Heijde scoring; UA = undifferentiated arthritis.

Assessing data on the total available duration of follow up, implying that patients with differences in the duration of follow up are compared, revealed that 23 UA patients (4.4%) developed RA later than one year after inclusion. Categorizing these patients in the RA group and using the cut off of having at least two erosive joints revealed a slightly higher PPV of 60% (95% CI 50% to 71%) and a slightly lower NPV of 69% (95% CI 65% to 74%).

### Effect of localization of erosion

Baseline erosions were present in the hands in 11.2% of UA patients, in the feet in 8.9% of UA patients, and both in hands and feet in 8.5% of UA patients (Table 1). From these data, it cannot be concluded that the small joints in the feet are less often erosive than the small joints in the hands because the number of assessed joints in feet and hands are different, 12 versus 26, respectively. Moreover, the LR+ for erosive joints in the feet is for all cut-off points somewhat higher as compared with the LR+ for erosive joints in the hands with a similar LR- (Tables 4 and 5). These data suggest that presence of erosions in the joints of the feet is slightly more predictive than erosions in the hand joints.

**Table 4:** Predictive value for the progression from UA to RA within one year using different cut-off values for erosions in hands

Number of erosive joints	n	PPV	(95% CI)	NPV	(95% CI)	Specificity	(95% CI)	Sensitivity	(95% CI)	LR+	LR-	AUC (SEM)
**≥1**	103	44	34-53	72	68-77	84	80-88	28	21-35	1.7	0.86	0.56 (0.028)
**≥2**	38	55	40-71	71	67-75	95	93-98	13	8-18	2.8	0.91	0.54 (0.028)
**≥3**	18	56	33-79	70	66-74	98	96-99	6	3-10	2.8	0.96	0.52 (0.028)
**≥4**	9	67	36-98	70	66-74	99	98-100	4	1-7	4.5	0.97	0.52 (0.028)
**≥5**	7	71	38-100	70	66-74	99	99-100	3	0-6	5.6	0.97	0.51 (0.028)

n = number of UA patients positive for this cutoff.

AUC = area under the curve; CI = confidence interval; LR+ = positive likelihood ratio; LR- = negative likelihood ratio; NPV = negative predictive value; PPV = positive predictive value; RA = rheumatoid arthritis; SEM = standard error of measurement; UA = undifferentiated arthritis.

**Table 5:** Predictive value for the progression from UA to RA within one year using different cut-off values for erosions in feet

Number of erosive joints	n	PPV	(95% CI)	NPV	(95% CI)	Specificity	(95% CI)	Sensitivity	(95% CI)	LR+	LR-	AUC (SEM)
**≥1**	89	49	39-60	73	69-77	87	84-91	28	21-34	2.2	0.83	0.57 (0.028)
**≥2**	42	60	48-74	72	68-76	95	93-98	16	10-21	3.3	0.89	0.55 (0.028)
**≥3**	18	67	45-88	70	66-74	98	97-100	8	3-12	4.5	0.94	0.53 (0.028)
**≥4**	9	67	36-98	70	66-74	99	98-100	4	1-7	4.5	0.97	0.52 (0.028)
**≥5**	5	80	45-115	70	66-74	100	99-100	3	0-5	8.9	0.98	0.51 (0.028)

n = number of UA patients positive for this cutoff.

AUC = area under the curve; CI = confidence interval; LR+ = positive likelihood ratio; LR- = negative likelihood ratio; NPV = negative predictive value; PPV = positive predictive value; RA = rheumatoid arthritis; SEM = standard error of measurement; UA = undifferentiated arthritis.

The frequency of erosions (with a cut off of two or more erosive joints) in the MCP joints was 3.1% in those who developed RA compared with 2.2% in those who did not develop RA (*P *= 0.6, PPV = 38.5%, LR+ = 1.4, LR- = 0.99). Erosive PIP joints were present in 3.1% of the RA group compared with 1.1% of the non-RA group (*P *= 0.1, PPV = 55.6%, LR+ = 2.8, LR- = 0.98). The frequency of erosions in the wrist joints in the RA group was 4.4% compared with 1.1% in the non-RA group (*P *= 0.04, PPV = 63.6%, LR+ = 3.9, LR- = 0.97). Although the frequency of erosions in the MTP joints in the RA group was 14.4% compared with 3.4% in the non-RA group (*P *< 0.001, PPV = 67.6%, LR+ = 4.3, LR- = 0.88; Table 6).

**Table 6:** Predictive value for the progression from UA to RA within one year by location of erosive joints and cutoff of at least two erosive joints

Location of erosions	n	PPV	(95% CI)	NPV	(95% CI)	Specificity	(95% CI)	Sensitivity	(95% CI)	LR+	LR-	AUC (SEM)
**Wrist**	7	63.6	32-88	69.8	66-74	98.9	97-100	4.4	2-9	3.9	0.97	0.52 (0.028)
**MCP**	5	38.5	15-68	69.3	65-73	97.8	95-99	3.1	1-8	1.4	0.99	0.50 (0.028)
**PIP**	5	55.6	23-85	69.5	65-73	98.9	97-100	3.1	1-8	2.8	0.98	0.51 (0.028)
**MTP**	23	67.6	5-9	71.6	67-76	96.6	94-98	14.4	10-21	4.3	0.88	0.56 (0.028)

n = number of UA patients positive for this cutoff.

AUC = area under the curve; CI = confidence interval; LR+ = positive likelihood ratio; LR- = negative likelihood ratio; MCP = metacarpo-phalangeal joint; MTP = metatarso-phalangeal joint; NPV = negative predictive value; PIP = proximal inter-phalangeal joint; PPV = positive predictive value; RA = rheumatoid arthritis; SEM = standard error of measurement; UA = undifferentiated arthritis.

### Characteristics of erosive UA patients that did not develop RA

As we observed that 47% of the UA patients with at least two erosive joints did not fulfill the 1987 ACR classification criteria for RA after one year of follow up, we compared the baseline characteristics of the patients who had at least two erosive joints that did and did not progress to RA (Table 7). The erosive patients that did not develop RA were less often anticyclic citrullinated peptide antibody positive (*P *< 0.001), less often RF positive (*P *= 0.01), had a lower erythrocytic sedimentation rate (*P *= 0.004), a lower C-reactive protein (*P *= 0.005), a lower tender joint count (*P *< 0.001), a lower swollen joint count (*P *< 0.001) and experienced less severe morning stiffness as recorded on a visual analogue scale (*P *= 0.04) compared with the patients that progressed to RA.

**Table 7:** Baseline characteristics of the UA patients with erosions (≥2 erosive joints) that did and did not progress to RA within one year

Characteristic	Total (n = 83)	Developed RA (n = 44)	Did not develop RA (n = 39)	*P*
**Female gender n (%)**	44 (53)	26 (53.8)	18 (46.2)	0.2
**Age in years (Mean ± SD)**	61.5 ± 16	60.7 ± 16.4	62.5 ± 15.6	0.6
**Symptom duration at inclusion in months (Mean ± SD)**	6.5 ± 6.3	7.1 ± 7.4	5.7 ± 4.8	0.9
**Morning stiffness (Mean ± SD)**	74.9 ± 100.5	92.2 ± 114.4	55.4 ± 78.9	0.04
**Tender joint count (Mean ± SD)**	6.9 ± 5.8	9 ± 6.7	4.6 ± 3.7	<0.001
**Swollen joint count (Mean ± SD)**	4.7 ± 4	6 ± 4	3.2 ± 3.4	<0.001
**HAQ score (Mean ± SD)**	0.89 ± 0.74	0.9 ± 0.73	0.87 ± 0.75	0.9
**ESR (mm/hr) (Mean ± SD)**	34.7 ± 24.7	41.3 ± 24	27.2 ± 23.6	0.004
**CRP (mg/L) (Mean ± SD)**	27.8 ± 31.4	33.4 ± 30.4	21.5 ± 31.7	0.005
**RF positivity n (%)**	31 (37.3)	22 (50)	9 (23.1)	0.01
**ACPA positivity n (%)**	25 (32.5)	20 (51.3)	5 (13.2)	< 0.001
**SHS erosion score (mean ± SD)**	4.2 ± 3.4	4.7 ± 4.3	3.7 ± 1.8	0.4

ACPA = anticyclic citrullinated peptide antibody; CRP = C-reactive protein; ESR = erythrocytic sedimentation rate; HAQ = health assessment questionnaire; RA = rheumatoid arthritis; SD = standard deviation; SHS = Sharp/van der Heijde scoring; UA = undifferentiated arthritis.

Morning stiffness score on a 100-mm visual analogue score.

Of the UA patients with baseline erosions who developed RA, 74% were treated with DMARDs in their first year (compared with 34% of the erosive UA patients who did not develop RA) and 66% of non-erosive UA patients who developed RA were treated with DMARDs (compared with 21% of non-erosive UA patients that did not develop RA). This indicates that rheumatologists did not treat erosive patients more aggressively than non-erosive patients. DMARDs used were methotrexate (34.8%), hydroxychloroquine (29.3%), sulphasalazine (25%), combination of methotrexate and hydroxychloroquine or sulphasalazine (8.7%), or other DMARDs (2.2%).

### Contribution to the prediction rule for RA development

Recently, a prediction rule was published that estimates the risk for individual UA patients to progress to RA using nine commonly assessed clinical and serological variables [7]. Presence of erosions is not part of this rule. As we hypothesized that using different cut offs or a different definition for erosive joints (according to the SENS method) may affect the predictive ability of the prediction rule, data on the number of erosive joints were added to the logistic regression model with a backward selection procedure that was used to derive the prediction rule [7]. For all the different cut-off values used, the presence of erosive joints was not an independent predictor for RA development (data not shown), and thus this information did not improve the predictive ability of the model.

When using the prediction rule, there is a group of patients (25% of all UA patients) for whom no accurate prediction of RA development can be made. These patients have a prediction score between 6.0 and 8.0 and for these patients the PPV for RA development is 48% and the NPV is 52%. It was tested if the radiological data on erosions can serve as an extra predictive tool, improving the NPV and PPV for this specific group of UA patients (n = 139). When using the cut off of at least two erosive joints, the PPV was 56% and the NPV was 63% [see Additional data file 1]. Together these results indicate that using data on joint erosion disease does not result in an important further differentiation of the risk estimation for RA in patients with UA additive to the predictive ability of the known clinical and serological factors.

### Predictive accuracy for disease persistency

As the outcome measure of fulfilling the 1987 ACR criteria for RA might be subject to discussion (because these criteria were not designed to identify RA in an early phase) and to circular reasoning (because the presence of hand erosions are part of the ACR criteria), we also tested the ability of the presence of erosive joints to predict disease persistency, defined as the absence of sustained remission. For this analysis we used all available follow-up data, implying that the follow up was not similar for all patients studied. During the whole period of follow up, 39.6% (n = 205) of UA patients achieved clinical remission, while the remaining 60.4% (n = 313) had a persistent disease course (remained UA, developed RA or developed a disease other than RA). The PPV for having a persistent disease course gradually increased with higher cut-off values (Table 8). When using the cut-off value of having two or more erosive joints, the PPV was 68% and the NPV was 41%. These PPV are somewhat higher compared with the data on RA development according to the ACR criteria, but because of overlapping 95% CIs the differences were not significant. In addition to the finding that from all UA patients with two or more erosive joints 32.5% achieved sustained remission, it was observed that from all UA patients that achieved sustained remission, 13.2% already had at baseline at least two erosive joints. For all cut-off values for the number of erosive joints the positive and negative LRs were around one (Table 8).

**Table 8:** Predictive value for having persistent disease in UA patients using different cut-off values for number of erosive joints

Number of erosive joints	n	PPV	(95% CI)	NPV	(95% CI)	Specificity	(95% CI)	Sensitivity	(95% CI)	LR+	LR-	AUC (SEM)
**≥1**	148	65	57-73	41	36-46	75	69-81	31	26-36	1.2	0.93	53 (0.026)
**≥2**	83	68	57-78	41	36-46	87	82-92	18	14-22	1.4	0.95	52 (0.026)
**≥3**	50	70	57-83	41	36-45	93	89-96	11	8-15	1.5	0.96	52 (0.026)
**≥4**	24	68	48-86	40	36-44	96	93-99	5	3-8	1.3	0.99	51 (0.026)
**≥5**	15	73	51-96	40	36-44	98	96-100	4	2-6	1.8	0.98	51 (0.026)

n = number of UA patients positive for this cutoff.

Patients with remission 205 (39.6%).

Patients with persistent disease 313 (60.4%).

AUC = area under the curve; CI = confidence interval; LR+ = positive likelihood ratio; LR- = negative likelihood ratio; NPV = negative predictive value; PPV = positive predictive value; SEM = standard error of measurement; UA = undifferentiated arthritis.

## Discussion

The current study explored the prognostic value of the presence of erosive joints for RA-development (defined by fulfilment of the 1987 ACR classification criteria) and for having a persistent disease course in patients with recent-onset UA. As the primary aim was to determine the risk on these disease outcomes for individuals, so that the results are useful for clinical practice, we concentrated on the PPV and NPV and put less emphasis on the sensitivity and specificity, which provide information on the quality of the test. The LRs compare probabilities of true results to false results and as such also provide information on the test but not on absolute probabilities for individuals. The PPV and NPV are dependent on the prevalence of the disease in the population and therefore the results of the present study apply to recent-onset UA patients seen by rheumatologists.

We observed that in the presence of at least two erosive joints, the PPV for RA-development within one year was 53% and the PPV for persistent disease was 68%. The presence of one or more erosive joint had a lower PPV (45%) and the presence of three or more or four or more erosive joints was not more predictive compared with the presence of two or more erosive joints (PPV 54% versus 53%). Thus, a considerable proportion of UA patients with erosive joints do not progress to RA. Additionally, the LRs for disease persistency were around one, illustrating the low impact of the quantity of erosions for the likelihood of persistent disease. Although in patients with RA, the presence of baseline erosions is a potent predictor for a severe destructive disease course, the present data implicate that for personalized medicine in UA, information on the presence of erosions alone is inadequate to obtain optimal treatment decision making.

The present data revealed that the PPVs and NPVs for having erosions in the feet joints were at least equal to the predictive values for having erosions in the small joints of the hands, and from all joints studied the LR+ was the highest for the MTP joints. This is notable as in the 1987 ACR classification criteria for RA [6], the presence of erosions in hands are included but feet erosions are not. In line with a previous study [17], the current study suggests that the presence of feet erosions should form part of the classification criteria as well.

Recently, a prediction rule for RA development was developed and validated [7,8,18], and presence of erosions was not part of this rule. The data presented revealed that radiological data on erosions, regardless of its definition, does not importantly improve the predictive ability of the prediction rule that is based on common clinical and serological risk factors.

In order to have a similar duration of follow up for all studied UA patients, progression to RA was evaluated after one year. This might have introduced misclassification because patients may have fulfilled the ACR criteria later on in the disease. Therefore RA development was also recorded in the total available follow up (mean 8 ± 3 years). Of UA patients, 4.4% developed RA later than one year after inclusion. Taking these patients into consideration led to a slight increase of the PPV and decrease of the NPV although 95% CIs overlapped.

In the current study we evaluated erosions in anteroposterior x-rays of the hands and posteroanterior x-rays of the feet without additional planes. This is in accordance with clinical practice in the Netherlands and many other countries. The use of an extra plane could have increased the sensitivity to detect erosions, but at the costs of extra irradiation and financial costs.

Data about the value of erosions detected by other new imaging modalities such as sonography or magnetic resonance imaging (MRI) in UA population are scarce. Duer and colleagues investigated the value of MRI erosions in UA patients without erosions at conventional radiography and found that the presence of MRI erosions has a PPV of 50%, a NPV of 85%, a LR+ of 2.7 and a LR- of 0.5 for prediction of RA development [19]. Tamai and colleagues studied the predictive value of MRI erosions (without knowledge if these were radiographically visible) and observed a PPV of 81.5%, a NPV of 48%, a LR+ of 3.18 and a LR- of 0.78 [20].

Several studies have shown that the current ACR criteria are not well suited for establishing early RA [21-24]. In the current study, many UA patients had arthritis in few joints, were seropositive and had erosive joints. Because they did not fulfill the 1987 ACR criteria for RA at baseline they were classified as UA but not as RA. This may also illustrate that the 1987 ACR criteria are not sensitive to diagnose RA at an early stage and further implies the need for new classification criteria suitable for early RA. Moreover, determining the predictive values of baseline erosions for the development of RA according to the ACR criteria can lead to circular reasoning as hand erosions are part of the criteria. Therefore, we studied the predictive accuracy for having persistent disease as well. This is in line with previous studies which used persistent disease as an outcome measure in UA patients [24,25]. Although thorough investigation of the medical files taught us that the group of patients with persistent disease is heterogeneous and the available duration of follow up in which persistency/remission is assessed differed between patients, the predictive values for RA development and disease persistency were in the same range.

## Conclusions

In conclusion, the presence of erosions in small joints of the hands and feet in UA patients at first presentation gives a risk for RA development of 53% after one year and a risk for persistent disease of 68%. These data imply that in early UA baseline erosions are not always predictive for a poor disease outcome and are on their own insufficient to found treatment decisions.

## Abbreviations

ACR: American College of Rheumatology; AUC: area under the curve; CI: confidence intervals; DMARD: disease-modifying anti-rheumatic drug; EAC: Leiden Early Arthritis Cohort; ICC: intra-class correlation coefficient; Ig: immunoglobulin; IP: inter-phalangeal joint; LR: likelihood ratio; MCP: metacarpo-phalangeal joint; MRI: magnetic resonance imaging; MTP: metatarso-phalangeal joint; NPV: negative predictive value; PIP: proximal inter-phalangeal joint; PPV: positive predictive value; RA: rheumatoid arthritis; RF: rheumatoid factor; ROC: receiver operating characteristic; SDC: smallest detectable change; SENS: Simplified Erosion Narrowing Score; SHS: Sharp/van der Heijde scoring; UA: undifferentiated arthritis.

## Competing interests

The authors declare that they have no competing interests.

## Authors' contributions

MT scored the patients' radiographs, performed the statistical analysis and drafted the manuscript. TH participated in the design of the study and helped to draft the manuscript. DH participated in the design of the study and helped to draft the manuscript. AH participated in the statistical analysis, participated in the design of the study and participated in drafting the manuscript. All authors read and approved the final manuscript.

## Supplementary Material

Additional file 1 A Word file containing a table that lists the predictive values for rheumatoid arthritis (RA) development (within one year of follow up) in undifferentiated arthritis (UA) patients that scored six to eight in the Leiden prediction rule with different cut-off values for erosions. Click here for file
