# Supplementary material for: The prognostic value of baseline erosions in undifferentiated arthritis
Source: Arthritis Res Ther. 2009 Oct 15;11(5):R155. doi: 10.1186/ar2832 (PMC2787272; doi:10.1186/ar2832)
Supplement: Additional file 1 — A Word file containing a table that lists the predictive values for rheumatoid arthritis (RA) development (within one year of follow up) in undifferentiated arthritis (UA) patients that scored six to eight in the Leiden prediction rule with different cut-off values for erosions. [file ar2832-S1.doc]

**Addendum**

**Table I: Predictive values for RA development in UA patients (within 1 year) that scored 6-8 in the prediction rule for different cut-off values for erosions**

| **Nr of Erosive Joints** | **n** | **PPV** | **(95%CI)** | **NPV** | **(95%CI)** | **Specificity** | **(95%CI)** | **Sensitivity** | **(95%CI)** | **LR+** | **LR-** | **AUC (SEM)** |
| --- | --- | --- | --- | --- | --- | --- | --- | --- | --- | --- | --- | --- |
| **≥ 1** | 54 | 54 | 40-67 | 66 | 56-76 | 69 | 59-79 | 50 | 37-63 | 1.6 | 0.72 | 0,60 (0,049) |
| **≥ 2** | 32 | 56 | 39-73 | 63 | 53-72 | 83 | 75-91 | 31 | 19-43 | 1.8 | 0.83 | 0,57 (0,050) |
| **≥ 3** | 22 | 64 | 44-84 | 62 | 54-71 | 90 | 84-97 | 24 | 13-35 | 2.4 | 0.84 | 0,57 (0,050) |
| **≥ 4** | 11 | 55 | 25-84 | 59 | 51-68 | 94 | 89-99 | 10 | 3-18 | 1.7 | 0.95 | 0,52 (0,050) |
| **≥ 5** | 7 | 57 | 21-94 | 59 | 51-68 | 96 | 92-100 | 7 | 0-13 | 1.9 | 0.97 | 0,52 (0,050) |

**Total number in this group 139**

**Patients developed RA 58 (41.7%)**

**Patients did not develop RA 81 (58.3%)**
